# Automatic Diagnosis of Major Depressive Disorder Using a High- and Low-Frequency Feature Fusion Framework

**DOI:** 10.3390/brainsci13111590

**Published:** 2023-11-15

**Authors:** Junyu Wang, Tongtong Li, Qi Sun, Yuhui Guo, Jiandong Yu, Zhijun Yao, Ning Hou, Bin Hu

**Affiliations:** 1 School of Information Science and Engineering, Lanzhou University, Lanzhou 730000, China; wangjy22@lzu.edu.cn (J.W.); ttli2022@lzu.edu.cn (T.L.); sunq2023@lzu.edu.cn (Q.S.); 220220942541@lzu.edu.cn (J.Y.); 2 Gansu Provincial Key Laboratory of Wearable Computing, Lanzhou University, Lanzhou 730000, China; guoyh2022@lzu.edu.cn; 3 School of Mathematics and Statistics, Lanzhou University, Lanzhou 730000, China; 4 Medical Department, The Third People’s Hospital of Tianshui, Tianshui 741000, China; 5 School of Medical Technology, Beijing Institute of Technology, Beijing 100081, China; 6 CAS Center for Excellence in Brain Science and Intelligence Technology, Shanghai Institutes for Biological Sciences, Chinese Academy of Sciences, Shanghai 200031, China

**Keywords:** major depressive disorder, magnetic resonance imaging, multi-modal, deep learning, high and low frequencies, feature fusion

## Abstract

Major Depressive Disorder (MDD) is a common mental illness resulting in immune disorders and even thoughts of suicidal behavior. Neuroimaging techniques serve as a quantitative tool for the assessment of MDD diagnosis. In the domain of computer-aided magnetic resonance imaging diagnosis, current research predominantly focuses on isolated local or global information, often neglecting the synergistic integration of multiple data sources, thus potentially overlooking valuable details. To address this issue, we proposed a diagnostic model for MDD that integrates high-frequency and low-frequency information using data from diffusion tensor imaging (DTI), structural magnetic resonance imaging (sMRI), and functional magnetic resonance imaging (fMRI). First, we designed a meta-low-frequency encoder (MLFE) and a meta-high-frequency encoder (MHFE) to extract the low-frequency and high-frequency feature information from DTI and sMRI, respectively. Then, we utilized a multilayer perceptron (MLP) to extract features from fMRI data. Following the feature cross-fusion, we designed the ensemble learning threshold voting method to determine the ultimate diagnosis for MDD. The model achieved accuracy, precision, specificity, F1-score, MCC, and AUC values of 0.724, 0.750, 0.882, 0.600, 0.421, and 0.667, respectively. This approach provides new research ideas for the diagnosis of MDD.

## 1. Introduction

Major depressive disorder (MDD) is a prevalent mental health disorder that has a significant impact on both the individual and society [1]. It often presents as a severe and enduring depression that is accompanied by a variety of physical and mental symptoms. The utilization of clinical data and advanced imaging techniques in the investigation of depression [2,3] can aid healthcare professionals in achieving a precise diagnosis. Presently, imaging technology continues to advance at a rapid rate. Due to their non-invasive ability to provide a more comprehensive insight into the mechanistic abnormalities associated with disease pathology, both diffusion tensor imaging (DTI) and functional magnetic resonance imaging (fMRI) have become prominent tools in the field of MDD research and diagnosis. Specifically, DTI can illuminate the structural and anisotropic attributes of the brain’s white matter fibers by tracking the diffusion patterns of water molecules, which provides a more profound understanding of the brain’s communication system. Additionally, using BOLD signals for studying changes in brain function is one of the fundamental methods of fMRI.

Traditional machine learning techniques are capable of extracting information from pre-processed data sources, including gray matter (GM) [4] and functional connectivity (FC) matrices, among others, for disease diagnosis. Meanwhile, deep learning is particularly adept at the automated extraction of higher-level features and has demonstrated excellent performance across a range of computer vision tasks [5]. Deep learning has been extensively employed in various data feature extraction applications, including encompassing computed tomography (CT) [6], positron emission tomography (PET) [7], and magnetic resonance imaging (MRI). Moreover, deep learning models for multimodal data exhibit superior capabilities in capturing qualitative data features compared to unimodal approaches, and they offer robust model interpretability. For instance, Song et al. [8] designed multicenter and multichannel pooling GCN to diagnose Alzheimer’s disease using fMRI and DTI modalities, with an average classification accuracy of 93.05% in their binary classification tasks. Wang et al. [9] proposed an adaptive multimodal neuroimage integration (AMNI) framework for automatic MDD detection using both functional and structural MRI modalities, which demonstrated the effectiveness of the proposed method. While researchers make use of various modal features for disease diagnosis, there is often a missed opportunity to leverage cross-fusion between different scale features from different modalities, resulting in the potential oversight of valuable information.

Wang et al. [10] used the depth model 3D-Densenet for MDD diagnosis with only unimodal information from MRI. Gao et al. [11] proposed an attention-guided, unified deep learning framework using only local structural characteristics for classification. Marwa et al. [12] utilized shallow deep learning architecture to extract only local feature information from brain MRI for identifying a multi-class Alzheimer’s disease. However, they only considered local or global information. Jang et al. [13] proposed a spach transformer to accomplish image denoising for PET modalities using local and global information, but few modalities were involved.

It is observed that convolutional neural networks (CNN) [14] predominantly emphasize local receptive fields during convolution, which vary in texture, shape, and size across various features. CNN leverages its robust capability in extracting effective local information to further harness more intricate, high-frequency local details. Nevertheless, fully concentrating on the entire dataset can be challenging, potentially resulting in the loss of information pertaining to long-range dependencies. Transformers [15] with self-attention mechanisms can minimize this shortcoming to capture global low-frequency information about data. In medical imaging, high-frequency components often convey specific details and edge information, including features like the border of brain sulci and gyri, the subtle texture of the cerebral cortex, and more, while low-frequency components typically reflect information at a larger scale, including things like tissue distribution and brain morphology. Qiu et al. [16] fused long-range dependencies and global context information to alleviate the problem of over-smoothing and over-fitting. Qin et al. [17] found that long-range transformers have a great advantage in content selection. From a particular perspective, the transformer’s capability to extract information over extensive distances is showcased.

Recently, Su et al. [18] proposed a convolutional model of 3DMKDR of electroencephalogram (EEG) signals for depression disorder recognition. Teng et al. [19] proposed a transformer-based modeling approach for depression prediction. Nonetheless, their emphasis was confined to either low-frequency or high-frequency information, potentially neglecting the comprehensive explorations of data.

To address the issue of information loss attributed to the absence of either high-frequency or low-frequency data, we have introduced a cross-fusion, which harnesses multiple modalities to encode low- and high-frequency feature representations for MDD diagnosis. This approach strengthens the adversarial robustness of the extracted feature model. The model consists of three core components: the meta-high-frequency encoder, the meta-low-frequency encoder, and integrated learning. Specifically, the meta-high-frequency encoder, which consists of a simple fully convolutional network (SFCN) [20], is better able to extract the modality’s high-frequency information with fewer parameters. The meta-low-frequency encoder, comprising the 4-head attention and cls_token with positional encoding added (positional encoding has the ability to learn to differentiate between positions and cls_token serves as a learnable embedding vector, which is pre-encoded to end up with a feature vector that can be used for classification), proves more efficient and expeditious in extraction of the modality’s low-frequency information. Consequently, it endeavors to steer our model towards a more comprehensive exploration of both localized specific features and global structural characteristics. Additionally, we designed MLP for feature extraction of the FC matrix and designed the cross-fusion of all the extracted different features of different modalities to obtain a deeper feature representation. To delve deeper into understanding the information loss attributed to the constraints of high-frequency and high-frequency fusion, as well as low-frequency and low-frequency fusion, we tried to explore this phenomenon in greater detail. Finally, the ensemble learning voting idea was used for classification. Compared with individual modules, ensemble learning provided greater improvements in classification performance. We summarize our contributions as follows:

- We proposed a novel multi-modality deep learning framework for automatic diagnosis of MDD;
- We developed a feature extractor to mine global dependencies and local responses using transformer and CNN architectures, respectively;
- We designed an ensemble learning voting mechanism to obtain predictions.

The rest of this paper is organized as follows: Section 2 describes the source of the subjects’ data and preprocessing. Section 3 exhibits the proposed model and experimental details. Section 4 shows the ablation experiment and comparison with other deep learning models. Section 5 provides the results of this study, limitations, and future improvements. Section 6 presents a summary.

## 2. Material

### 2.1. Subjects

We collected information on three modalities—DTI, fMRI, and sMRI—from 128 participants, and all patients with MDD in this study received a clinical diagnosis based on the structured clinical interview for diagnostic and statistical manual of mental disorders, fourth edition (DSM-IV) axis i disease (SCID). HCs (healthy controls) were recruited using the non-patient edition of the structured clinical interview for DSM-IV.

All participants were within the age range of from 18 to 65 and did not manifest any other mental illnesses. Furthermore, we obtained approval from the Ethics Committee of Gansu Provincial Hospital, China (Approval No. 2017-071, 6 July 2017). Prior to participation, individuals provided informed consent after attaining a comprehensive understanding of the study’s objectives, potential risks, and benefits.

### 2.2. Data Processing

The rs-fMRI images underwent preprocessing, utilizing the unified data processing assistant for the resting-state fMRI (DPARSF) pipeline within the DPARSF V6.2_220915 toolbox [21]. These preprocessing steps primarily encompassed head motion correction, slice timing correction, spatial normalization, and spatial smoothing. We proceeded to extract the time series data from 116 brain regions using the automated anatomical labeling (AAL) templates. Subsequently, by calculating the Pearson correlation coefficients between pairs of these brain regions, we derived the final FC matrix.

We applied the PANDA 1.3.1 software (http://www.nitrc.org/projects/panda) to preprocess the raw DTI data. Ultimately, the fractional anisotropy mapping (FAM) was generated by mapping from the MNI space to the AAL template.

For sMRI, we used the CAT12 toolbox (http://dbm.neuro.uni-jena.de/vbm/) implemented in the SPM12 software (http://www.fil.ion.ucl.ac.uk/spm/) to extract normalized gray matter volumes.

Following data preprocessing, the data size of the FAM was 91 × 109 × 91, while the size of the sMRI gray matter image was 113 × 137 × 113. To match the model inputs, we used simpleITK (simpleITK is an open source tool library for medical image processing) in the sitkNearestNeighbor to modify the size of the input data.

The clinical diagnostic characteristics of the participants are shown in Table 1. Excessive head movement (rotation degree > 2°, translation distances > 2 mm, or mean FD (Jenkinson) > 0.2) and missing modalities were excluded from the analysis. Patients clinically diagnosed with MDD and possessing HAMD scores > 7 were included. A total of 116 subjects were eventually further analyzed, including 54 MDDs and 62 HCs.

Following the processing, we obtained a 3D medical image size of 112 × 112 × 112 for both FAM and sMRI, while the FC matrix size from fMRI remained unchanged at 116 × 116. As a final step, we introduced a minute value of 1 × 10^−9^ to normalize all the data, thereby preventing division by zero.

## 3. Methods

This paper introduces an approach that integrates both CNN and transformer architectures to extract features encompassing global low-frequency information and local high-frequency information and then fuses these features.

### 3.1. Overview

**Figure 1:**
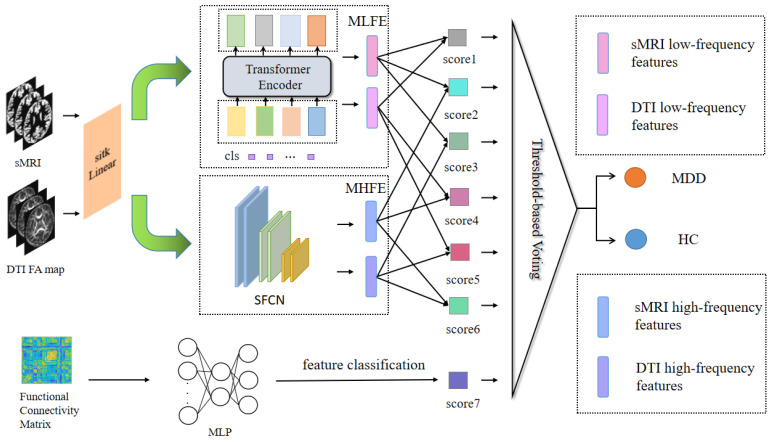
Overall structure of the proposed model. Abbreviations: MLFE = meta-low-frequency encoder, MHFE = meta-high-frequency encoder, MDD = major depressive disorder, HC = healthy control.

The proposed model primarily consisted of encoders for extracting high- and low-frequency features. These encoders encompassed the meta-low-frequency encoder (MLFE) and the meta-high-frequency encoder (MHFE). The MLFE was designed as an encoder for extracting low-frequency information, adapted to capture features in medical images that encapsulate global information. This proficiency was valuable for comprehending the overarching characteristics of the data. Conversely, MHFE was designed as an encoder to extract high-frequency depth features from the image. These features represented the local key attributes of the image, enabling the removal of redundant information and the representation of a unique and stable data structure to a significant extent. Additionally, the model incorporated a MLP for the extraction of functional features from the FC matrix, as illustrated in Figure 1.

### 3.2. Meta-Low-Frequency Encoder

**Figure 2:**
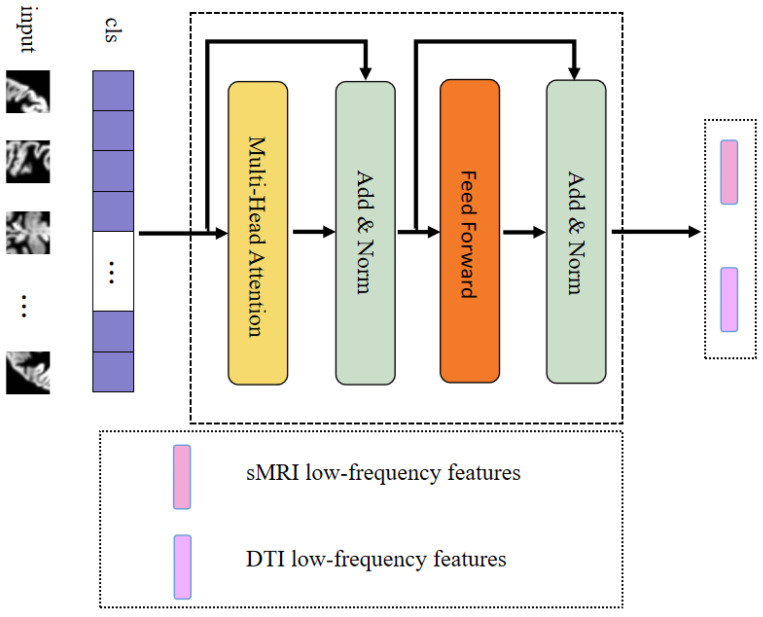
Meta-low-frequency encoder.

Low-frequency information typically signifies slowly evolving structural characteristics and global patterns, corresponding to alterations occurring over longer spatial or temporal scales. This enables the capture of macroscopic brain structural features. In this module, we devised the meta-component for low-frequency feature extraction responsible for acquiring low-frequency feature information from FAM and sMRI, as depicted in Figure 2. The transformer encoder [22] element served as the foundation for our design in this module.

To enhance computational efficiency, we selected 4 heads of attention in the transformer encoder, set the individual word vector to 512, and set num_layers to 6. This method was utilized to develop lightweight models, which were useful for implementing models in resource-constrained situations and could improve model utility. Initially, we generated a positional encoding vector for the cls_token in a random manner. Prior to this, we selected a convolutional layer rather than a linear layer to boost the module’s performance, and finally, positional encoding was added to the input data.

Through the implementation of MLFE, we could subsequently acquire information pertaining to the low-frequency features within the corresponding modalities.

### 3.3. Meta-High-Frequency Encoder

High-frequency information typically conveys localized details with rapidly changing characteristics, corresponding to changes on shorter spatial or temporal scales. This makes local subtleties and minute changes in the brain’s architecture easier to capture. In this module, we designed the meta-module, which was made up of SFCN to extract sMRI and FAM high-frequency features.

**Figure 3:**
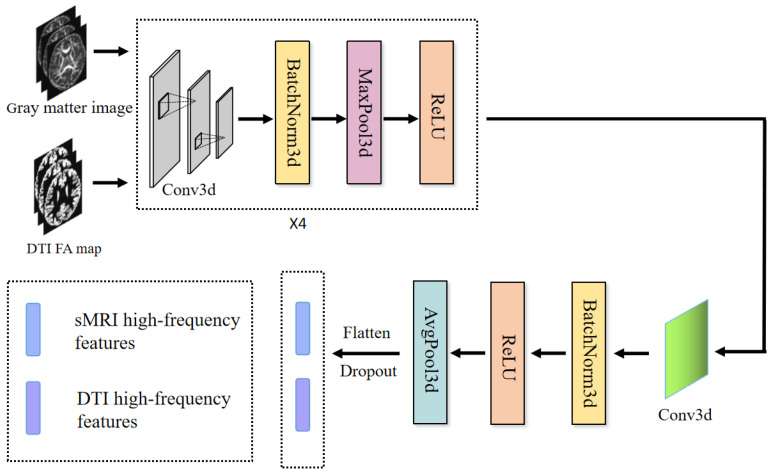
Meta-high-frequency encoder.

This module comprised a convolutional layer in combination with an average pooling layer. The channel sizes of the convolutional layers were configured as [32, 64, 128, 64, 32]. Notably, the last layer did not contain a max-pooling operation and used a 1 × 1 × 1 convolutional kernel, whereas all the previous layers contained a max-pooling layer and a 3 × 3 × 3 convolution with a padding value of 1. Then, it went through the sequence of convolutional, BatchNorm, and ReLU layers, ending with the average pooling layer, shown in Figure 3.

We then could acquire high-frequency data describing the modalities’ microscopic characteristics using MHFE.

### 3.4. Multilayer Perceptron

To obtain the FC matrix information, we first calculated the integrating time series extracted from the fMRI, which could reveal the internal functional characteristics of the brain, and help to better obtain useful information and explore the difference between disease and normal state. Finally, we let the FC matrix go through MLP for further analysis.

This module extracted the high-level abstract FC matrix features from fMRI. The MLP included an input layer, a hidden layer, and an output layer. Each layer was accompanied by a ReLU activation layer and a dropout layer with a rate of 20%. The final output consisted of a single logit value obtained from the MLP.

### 3.5. Feature Fusion

We fused the extracted sMRI and FAM, corresponding to the low-frequency features and high-frequency features, respectively, according to the high-high-frequency fusion, low-low-frequency fusion, and high-low-frequency fusion. The micro- and macro-features of each modality could be extracted via high-high-frequency fusion and low-low-frequency fusion. High-low-frequency fusion serves as compensation for the potential loss of features in each modality encountered in the initial two approaches. We amalgamated the six features using three feature fusion methods, which proved more effective in capturing the potential interactions between multimodal sources and within each modality. As a result, we obtained six logit values corresponding to the fusion process.

The six logit values, along with the single logit value extracted from the fMRI data features, were each subjected to a sigmoid activation layer to yield the seven values essential for the final voting process.

### 3.6. Ensemble Learning Voting

Ensemble learning seeks to enhance a model’s performance and stability by combining predictions from multiple weak learners. This approach mitigates the risk of overfitting, boosts the model’s generalization capabilities, enhances its robustness, and ultimately leads to more precise prediction or classification outcomes. Furthermore, ensemble learning helps diminish misclassification attributed to data noise or uncertainty.

Each model in this approach predicted the sample and then this was compared to the threshold we set. The final prediction was determined through the use of the majority vote principle.

### 3.7. Experiment Detail

The experiments were compiled with pytorch-1.8.2 and run on GPUs of NVIDIA Tesla V100 based on Ubuntu 18.04. The model was trained for a number of 200 epochs, utilizing a binary cross-entropy (BCE) loss function with a small batch size of 4. We used the Adam optimizer [23] with a learning rate of 9 × 10^−4^ and a weight decay of 1 × 10^−8^. To evaluate the model’s performance, we implemented a 4-fold cross-validation on the dataset, partitioning the data into four subsets. In each fold, one subset served as the testing set, while the other three subsets were utilized for model training. Ultimately, the mean ± SD was used as the result.

### 3.8. Evaluation Metrics

The accuracy (ACC) (Equation (1)), precision (PREC) (Equation (2)), recall (REC) (Equation (3)), specificity (SPE) (Equation (4)), F1-score (F-1), Matthew’s correlation coefficient (MCC), and area under the receiver operating characteristic (ROC) curve (AUC) were used to evaluate classification performance,

(1) $$ACC=\frac{TP+TN}{TP+TN+FP+FN}$$

(2) $$PREC=\frac{TP}{TP+FP}$$

(3) $$\;REC=\frac{TP}{TP+FN}$$

(4) $$\;SPE=\frac{TN}{TN+FP}$$

(5) $$\;F-1=2\times \frac{PREC\times REC}{PREC+REC}$$

(6) $$\;MCC=\frac{TN\times TP-FN\times FP}{\sqrt{\left(TN+FP)(FN+TP)(TN+FN)(TP+FP\right)}}$$

where TP, FN, FP, and TN represent True Positive, False Negative, False Positive, and True Negative, respectively.

## 4. Results

In this section, we set up ablation experiments as well as comparisons with others with the aim of verifying the validity of our proposed models. These comparison experiments included experiments with individual modal combination situations as inputs, experiments with high- and low-frequency sub-modules, and experiments comparing classical CNN as well as transformer models. To ensure the reliability of our results, we used the same standard for dividing the datasets in all experiments. We non-overlappingly divided the datasets into the training set (87) and the test set (29) in a ratio of 3:1, where the training set was used to train the weights of the models and the test set was used to test the models.

### 4.1. Ablation Experiments

To validate the robustness of the model, we systematically deconstructed it and analyzed its components individually. First, we assessed the cross-fusion of various modalities by validating the performance of each modality in isolation and in various paired combinations. The data size division used in this experiment remained the same as above. All assessment indicators were consistent, and the assessed results are presented in Table 2.

Next, we validated the fusion effect of different low- and high-frequencies. The division of the data for this experiment remained as previously described. Then, we validated the three modules of low- and high-frequency and fMRI blocks, respectively, so as to verify the advantages of the proposed fusion method, and the evaluation metrics are shown in Table 3.

### 4.2. Comparison with Other Models

In this section, a comprehensive comparison was made between the proposed model and four currently popular deep learning models (LeNet, ResNet, DenseNet, and Vision Transformer). In this experiment, we used the same data size division as before. Using the same model settings, the purpose of this comparison was to evaluate the validity of the proposed model. Table 4 shows further details. The proposed model extracted data features more comprehensively and performed feature fusion differently from other models for the extracted features, which was advantageous to the final classification diagnosis.

## 5. Discussion

MDD is a complex and common disorder with an uncertain cause. Deep learning models for the diagnosis of MDD have been widely proposed with the advancement of medical imaging technology and algorithms. However, previous studies have mostly concentrated on single-scale modal feature data used as disease diagnostic criteria and have overlooked the possible influence of cross-fusion between various modalities. Simultaneously, during the modal feature extraction process, a singular focus on either local high-frequency or global low-frequency information is prevalent. Traditional fusion techniques employed in these situations may inadvertently mask potential interactions between high- and low-frequency information. As a result, this may further reduce the available data features and ultimately diminish the effectiveness of the model in disease diagnosis. Thus, our completed experiments substantiated significantly improved results when employing multimodal input for extracting high- and low-frequency features, as opposed to using fewer modalities for this purpose. These results could be attributed to the broader representational capacity of multimodal data and the enhanced utilization of valuable information. Furthermore, the results derived from the exclusive use of high- or low-frequency fusion techniques exhibited substantial differences when compared to the results obtained through the three fusion methods for high- and low-frequency. This discrepancy underscored the idea that the effective integration of high- and low-frequency features yields more favorable diagnostic results.

We compared current approaches for diagnosing MDD based on deep learning models. Zhu et al. [25] proposed the only deep graph convolutional neural network (DGCNN) method for brain network classification between 830 MDD patients and 771 normal controls (NC), with a final accuracy of 72.1%. Venkatapathy et al. [26] proposed an ensemble model for the classification between 821 patients with MDD and 765 HCs, and the final model achieved 71.18% accuracy in upsampling and 70.24% accuracy in downsampling. Hu et al. [27] proposed a transformer-based BrainNPT model for brain network classification on a large dataset of REST-meta-MDD, and the accuracy of the model after pre-training reached 70.25%. The reason that these models are less accurate than ours is likely due to the focus on more particular details or the reality that the long- and short-distance information are not sufficiently mined for fusion, even though the amount of this data is much larger than ours.

The integration of high- and low-frequency information represents a crucial approach in clinical diagnosis, encompassing image features across various scales and providing a robust foundation for disease diagnosis and analysis. Specifically, the extraction of the high-frequency component in images is concentrated on the intricate details within the image. These details are essential for identifying diseases because they assist in recognizing subtle changes in pathology. Conversely, the extraction of low-frequency components in images characterizes the macroscopic structures and features that exist within the image. This global perspective complements the local details, providing a vital component of information that proves critical in the final evaluation of the disease. High-frequency and low-frequency information can have distinct features in a variety of medical problems. This integrated method enables healthcare practitioners to selectively emphasize important components, allowing them to conduct a full assessment that easily moves from micro to macro and vice versa. This comprehensive evaluation improves their ability to determine the patients’ health status, evaluate therapy outcomes, and develop a more personalized treatment strategy.

We proposed a model for cross-fusion of multimodal features based on high and low frequency, aiming at a better and more thorough utilization of high and low frequency information and an effective resolution of the prior issue. In the case of high- and low-frequency features, the fusion of high-frequency and low-frequency data presented a distinct perspective compared to other feature information. This approach aims to comprehensively bridge the gaps between the overlooked features, gain a deeper understanding of feature interactions, and enhance the diagnosis of MDD. The addition of our cross-fusion method to a previous fusion scheme fully explored this further and made up for the missing information between the neglected features and achieved a more comprehensive feature interaction.

We believe that the proposed model is of great generalization and migration ability. Although our study focuses on specific disease detection, the essential principles and approaches of the model are applicable to other medical image-based disease diagnoses. We believe that if the structural properties of the data are similar, the model can produce similar results in related domains. However, every domain faces its own set of challenges that need adaptation and validation for better use in other fields. Future study might investigate the model’s potential for use and evaluation in other fields.

Although our model achieved satisfactory results, there are still some shortcomings. On the one hand, the dataset we used was relatively small, and the size of the dataset affects the effect of the deep learning model to a certain extent. On the other hand, we only used the more common modal features as inputs to the model, and whether there are other features that can further improve the classification ability of our model needs to be further verified.

In future research, we aim to enhance the diagnostic efficacy of the model through the pursuit of two key avenues. (1) Enriching modal data information: our goal is to add the number of modalities of the data to improve the diversity and quality of the data. The work being performed will allow for a deeper and more comprehensive understanding of the features of the illness. (2) Enhancing encoder design: our goal is to design a more efficient encoder that can quickly, accurately, and deeply extract underlying data features. This enhancement will elevate the quality of features deployable in disease diagnosis.

## 6. Conclusions

In this paper, we proposed a multimodal cross-fusion MDD diagnostic model based on high- and low-frequency information. We designed MHFE and MLFE to capture more profound local high-frequency and global low-frequency information from multimodal magnetic resonance imaging data. By cross-fusing these extracted features, we aimed to address the issue of potential feature loss. Upon extracting the profound functional features from the FC matrix through the MLP, we uniformly classified them utilizing the ensemble learning voting strategy. This approach has the potential to enhance classification performance beyond that of a single module. The model achieved a 72.4% accuracy rate, which highlighted the necessity to study the interactions between multimodal high and low frequencies information.

## Figures and Tables

**Table 1:** The clinical diagnosis characteristics of the participants.

	MDD	HCs
Number of participants	54	62
HAMA	17.19 ± 7.58	-
HAMD (17-item)	17.62 ± 5.95	-

Abbreviations: MDD = Major Depressive Disorder, HCs = healthy controls, HAMA = Hamilton anxiety scale, HAMD = Hamilton depression rating scale.

**Table 2:** Comparisons of different multi-modal inputs in the proposed model.

Modalities	ACC	PREC	REC	SPE	F-1	AUC	MCC
sMRI	0.620 ± 0.088	0.550 ± 0.043	0.500 ± 0.121	0.710 ± 0.082	0.520 ± 0.101	0.667 ± 0.023	0.209 ± 0.021
fMRI	0.517 ± 0.061	0.444 ± 0.068	0.667 ± 0.042	0.412 ± 0.116	0.533 ± 0.038	0.593 ± 0.042	0.080 ± 0.042
DTI	0.517 ± 0.052	0.400 ± 0.031	0.333 ± 0.153	0.647 ± 0.095	0.364 ± 0.069	0.618 ± 0.061	−0.020 ± 0.066
sMRI + fMRI	0.690 ± 0.079	0.667 ± 0.074	0.500 ± 0.086	0.824 ± 0.112	0.571 ± 0.076	0.711 ± 0.077	0.344 ± 0.115
sMRI + DTI	0.655 ± 0.044	0.583 ± 0.069	0.583 ± 0.098	0.706 ± 0.137	0.583 ± 0.124	0.642 ± 0.054	0.289 ± 0.023
fMRI + DTI	0.586 ± 0.056	0.500 ± 0.049	0.500 ± 0.078	0.647 ± 0.063	0.500 ± 0.073	0.652 ± 0.035	0.147 ± 0.121
sMRI + fMRI + DTI	0.724 ± 0.021	0.750 ± 0.028	0.500 ± 0.054	0.882 ± 0.044	0.600 ± 0.034	0.667 ± 0.029	0.421 ± 0.033

**Table 3:** Comparison of different branches of the proposed model using sMRI, fMRI, and DTI as inputs.

Models	ACC	PREC	REC	SPE	F-1	AUC	MCC
MHFE	0.690 ± 0.053	0.670 ± 0.048	0.500 ± 0.031	0.820 ± 0.065	0.570 ± 0.074	0.650 ± 0.024	0.344 ± 0.011
MLFE	0.517 ± 0.023	0.438 ± 0.034	0.583 ± 0.029	0.471 ± 0.053	0.500 ± 0.062	0.542 ± 0.051	0.053 ± 0.213
Only fMRI block	0.517 ± 0.061	0.444 ± 0.068	0.667 ± 0.042	0.412 ± 0.116	0.533 ± 0.038	0.593 ± 0.042	0.080 ± 0.042
Proposed model	0.724 ± 0.021	0.750 ± 0.028	0.500 ± 0.054	0.882 ± 0.044	0.600 ± 0.034	0.667 ± 0.029	0.421 ± 0.033

**Table 4:** Comparison of different encoders between the proposed model and classical CNN models using sMRI, fMRI, and DTI as inputs.

Models	ACC	PREC	REC	SPE	F-1	AUC	MCC
LeNet *	0.620 ± 0.028	0.533 ± 0.022	0.667 ± 0.041	0.588 ± 0.048	0.593 ± 0.067	0.662 ± 0.049	0.251 ± 0.022
ResNet *	0.621 ± 0.042	0.545 ± 0.039	0.500 ± 0.047	0.706 ± 0.053	0.522 ± 0.035	0.613 ± 0.052	0.209 ± 0.041
DenseNet *	0.655 ± 0.012	0.583 ± 0.055	0.583 ± 0.102	0.706 ± 0.076	0.583 ± 0.042	0.637 ± 0.023	0.289 ± 0.053
Vision Transformer [24] *	0.552 ± 0.089	0.467 ± 0.042	0.583 ± 0.057	0.529 ± 0.039	0.519 ± 0.085	0.500 ± 0.097	0.111 ± 0.037
Proposed model	0.724 ± 0.021	0.750 ± 0.028	0.500 ± 0.054	0.882 ± 0.044	0.600 ± 0.034	0.667 ± 0.029	0.421 ± 0.033

Notes: * denotes classical deep learning model.

## Data Availability

The data presented in this study are available on request from the corresponding author.
